# Comprehensive Registry of Esophageal Cancer in Japan, 2011

**DOI:** 10.1007/s10388-018-0614-z

**Published:** 2018-04-18

**Authors:** Yuji Tachimori, Soji Ozawa, Hodaka Numasaki, Ryu Ishihara, Hisahiro Matsubara, Kei Muro, Tsuneo Oyama, Yasushi Toh, Harushi Udagawa, Takashi Uno, The Registration Committee for Esophageal Cancer of the Japan Esophageal Society

**Affiliations:** 1 Cancer Care Center, Kawasaki Saiwai Hospital, 31-27 Omiya-cho, Saiwai-ku, Kawasaki, Kanagawa 212-0014 Japan; 2 Department of Gastroenterological Surgery, Tokai University School of Medicine, Isehara, Japan; 3 Department of Medical Physics and Engineering, Osaka University Graduate School of Medicine, Osaka, Japan; 4 Department of Gastrointestinal Oncology, Osaka International Cancer Institute, Osaka, Japan; 5 Department of Frontier Surgery, Graduate School of Medicine, Chiba University, Chiba, Japan; 6 Department of Clinical Oncology, Aichi Cancer Center Hospital, Nagoya, Japan; 7 Department of Gastroenterology, Saku General Hospital, Nagano, Japan; 8 Department of Gastroenterological Surgery, National Kyushu Cancer Center, Fukuoka, Japan; 9 Department of Gastroenterological Surgery, Toranomon Hospital, Tokyo, Japan; 10 Department of Radiology, Graduate School of Medicine, Chiba University, Chiba, Japan

## Preface 2011

We deeply appreciate the great contributions of many physicians in the registry of esophageal cancer cases. The Comprehensive Registry of Esophageal Cancer in Japan, 2011, was published here, despite some delay. The registry complies with the Act for the Protection of Personal Information. The encryption with an HASH function is used for anonymity in an unlinkable fashion.

We briefly summarized the Comprehensive Registry of Esophageal Cancer in Japan, 2011. Japanese Classification of Esophageal Cancer 10th and UICC TNM Classification 7th were used for cancer staging according to the subjected year. A total of 6993 cases were registered from 300 institutions in Japan. Tumor locations were cervical: 4.5%, upper thoracic: 13.0%, middle thoracic: 47.8%, lower thoracic: 27.2%, and EG junction: 7.1%. Superficial carcinomas (Tis, T1a, and T1b) were 36.4%. For the histologic type of biopsy specimens, squamous cell carcinoma and adenocarcinoma accounted for 88.3 and 5.3%, respectively. Regarding clinical results, the 5-year survival rates of patients treated using endoscopic resection, concurrent chemoradiotherapy, radiotherapy alone, or esophagectomy were 86.0, 28.1, 26.5, and 54.5%, respectively. The endoscopic submucosal dissection accounted for 78.1% of endoscopic resection. Esophagectomy was performed in 4147 cases. Concerning the approach used for esophagectomy, 33.5% of the cases were treated thoracoscopically. The operative mortality (within 30 days after surgery) was 0.65% and the hospital mortality was 3.76%. The 5-year survival rate of patients with pStage IV in UICC classification (including patients with supraclavicular node metastasis) was better than that of patients with pStage IVb in JES classification (not including patients with supraclavicular node metastasis).

We hope that this Comprehensive Registry of Esophageal Cancer in Japan for 2011 will help to improve all aspects of the diagnosis and treatment of esophageal cancer in Japan.

## Contents

**Fig. 1:**
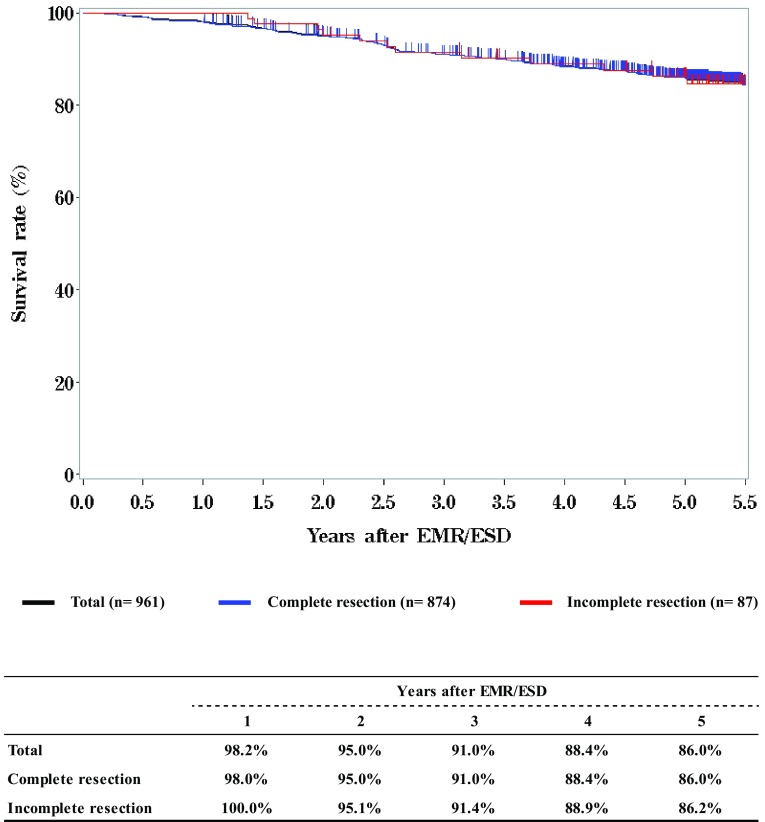
Survival of patients treated with EMR/ESD

**Fig. 2:**
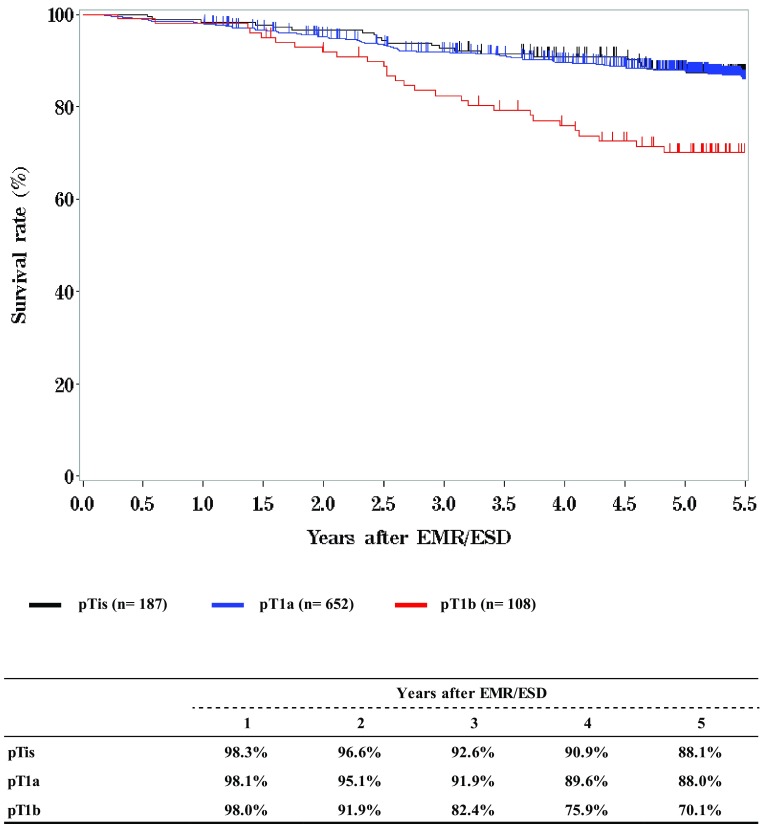
Survival of patients treated with EMR/ESD according to the pathological depth of tumor invasion (pT)

**Fig. 3:**
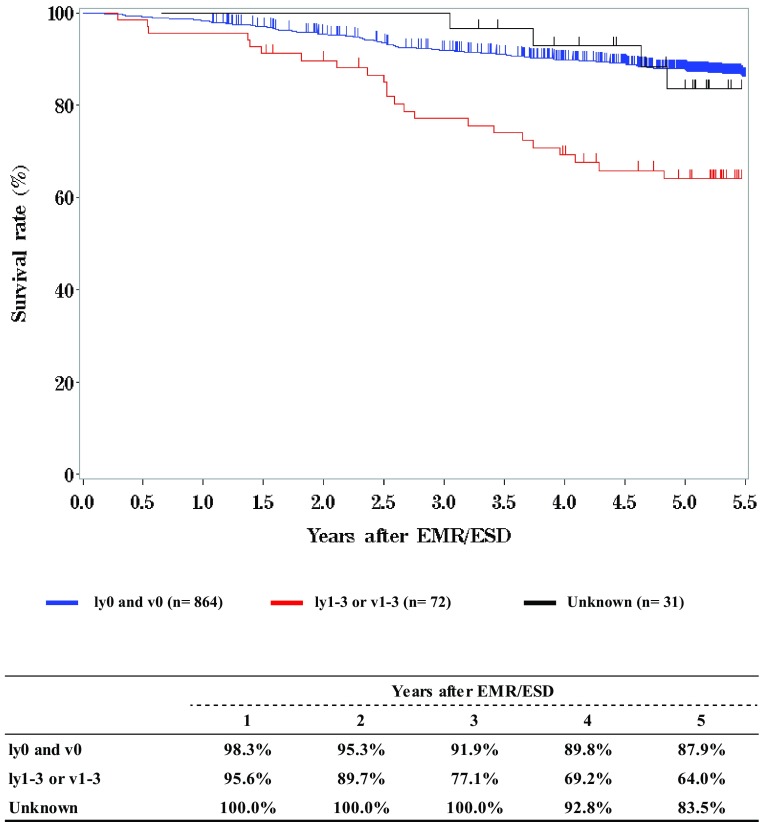
Survival of patients treated with EMR/ESD according to the lymphatic and venous invasion

**Fig. 4:**
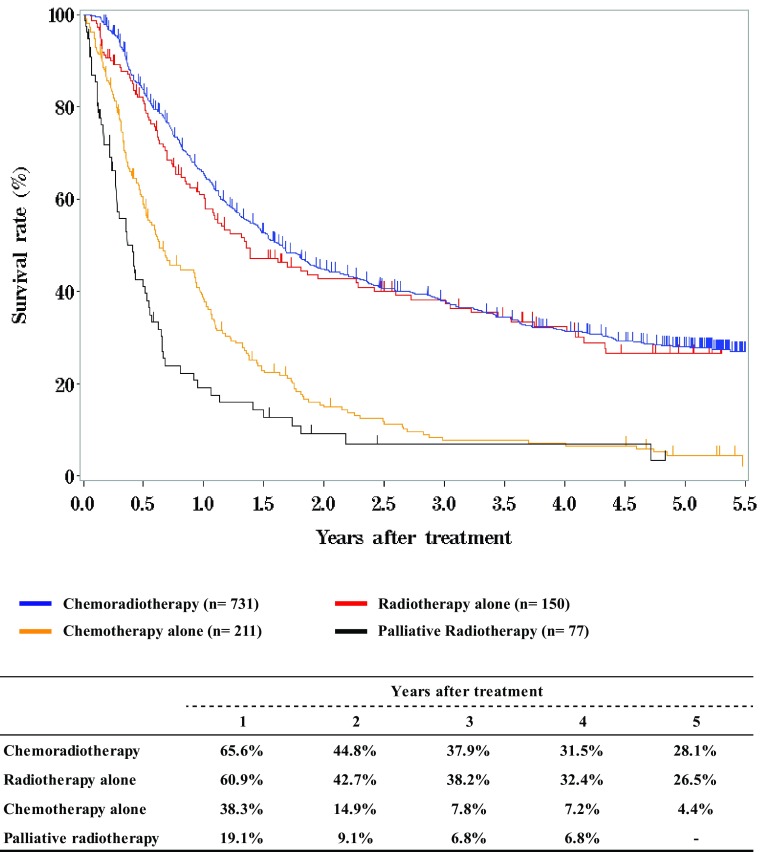
Survival of patients treated with chemotherapy and/or radiotherapy

**Fig. 5:**
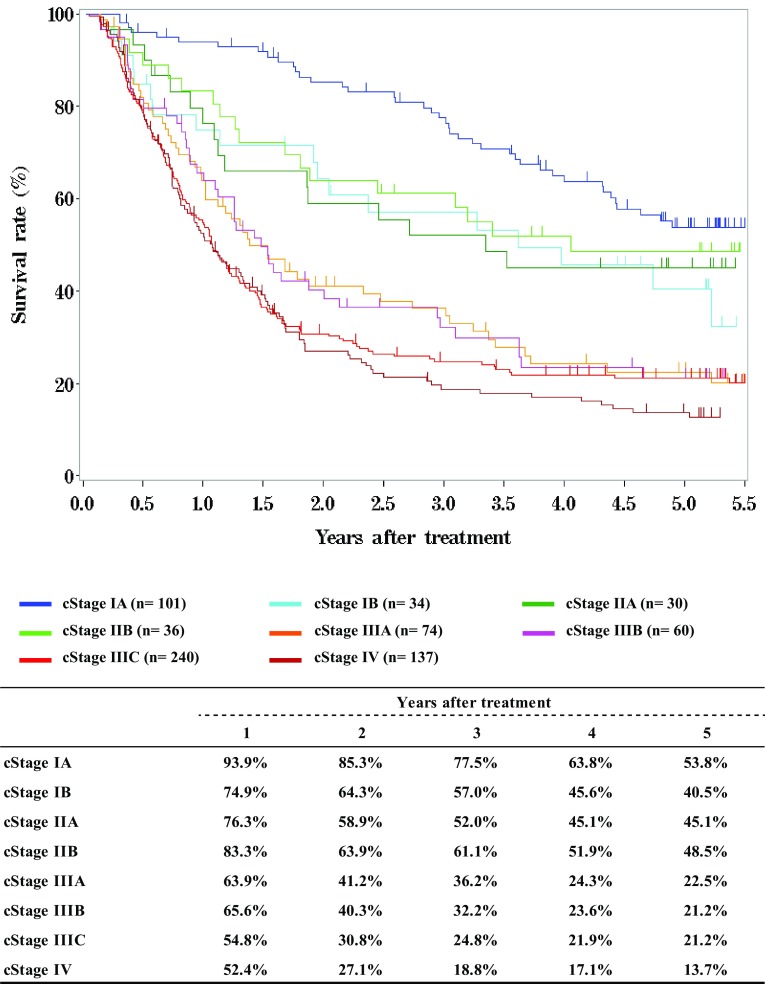
Survival of patients treated with definitive chemoradiotherapy according to clinical stage (UICC TNM 7th)

**Fig. 6:**
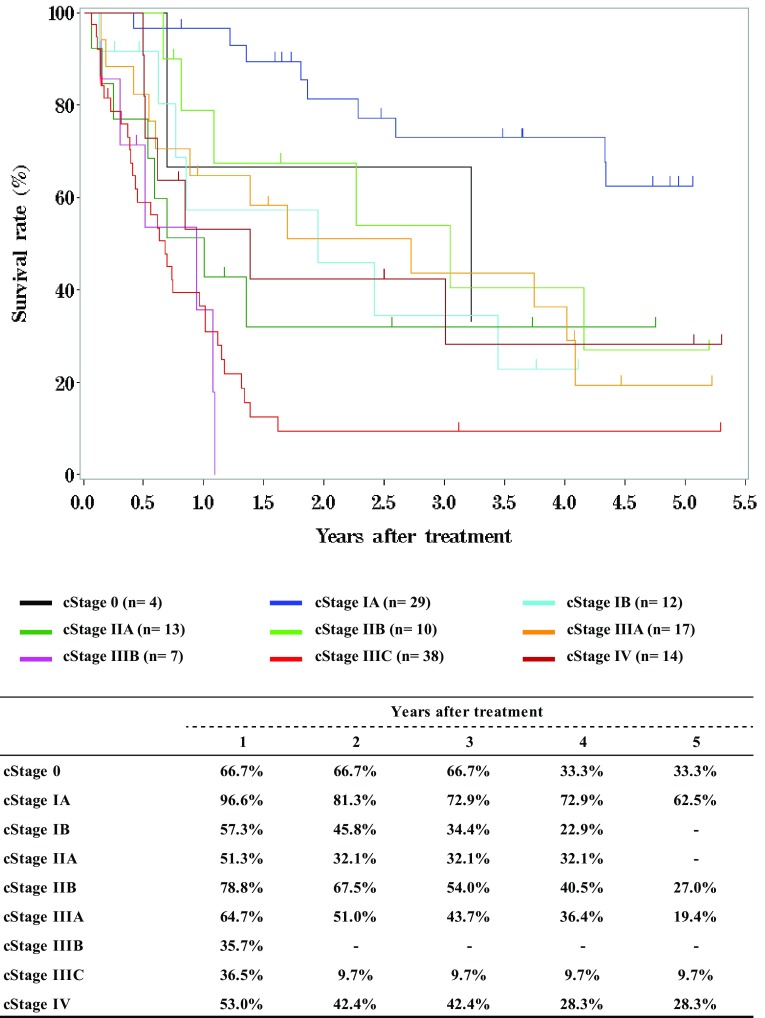
Survival of patients underwent radiotherapy alone according to clinical stage (UICC TNM 7th)

**Fig. 7:**
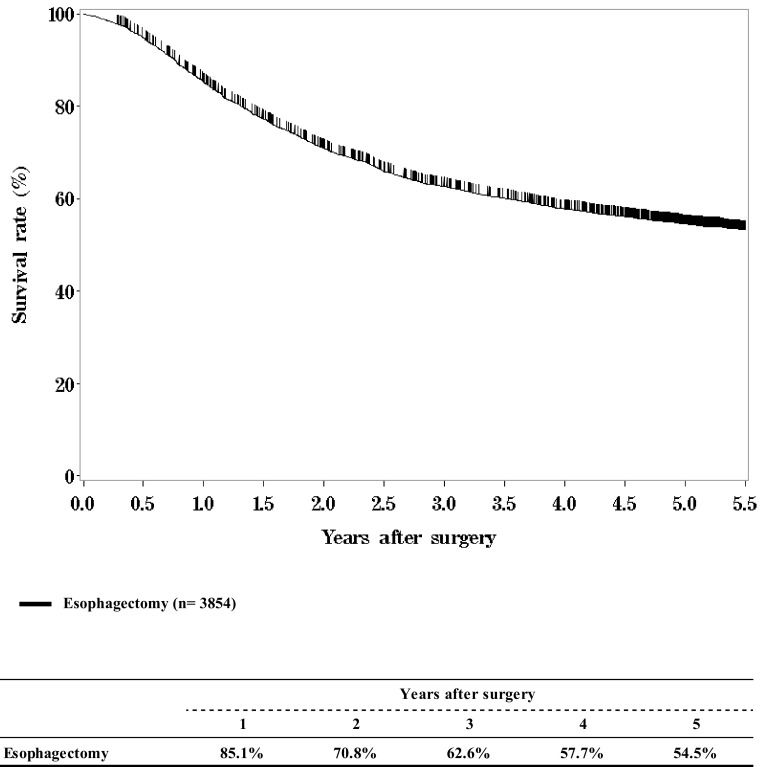
Survival of patients who underwent esophagectomy

**Fig. 8:**
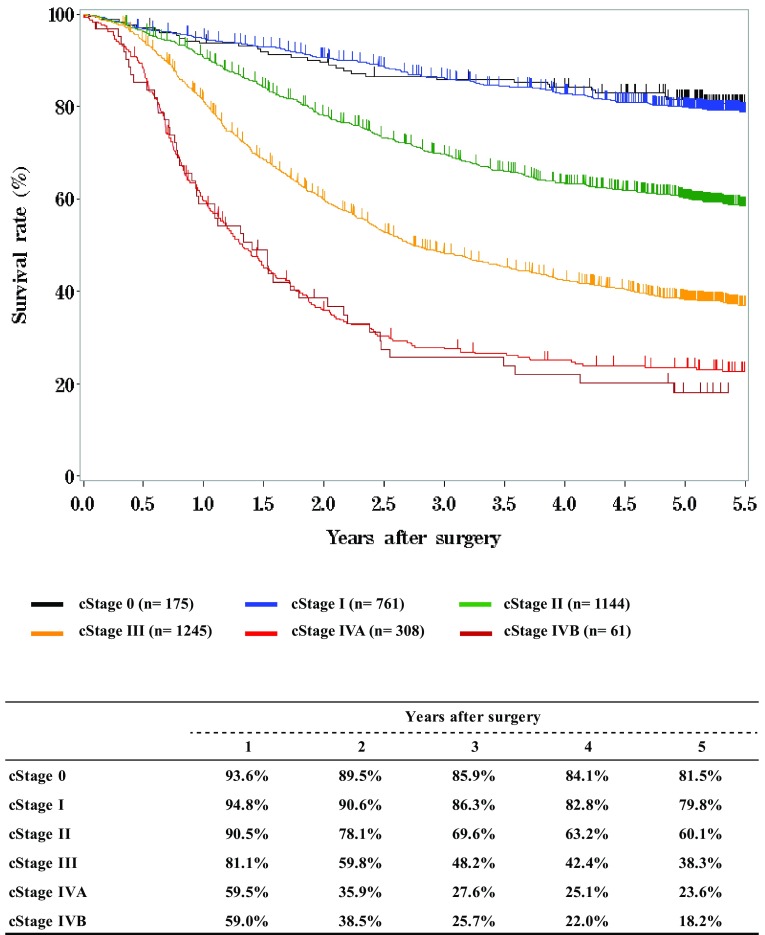
Survival of patients who underwent esophagectomy according to clinical stage (JES 10th)

**Fig. 9:**
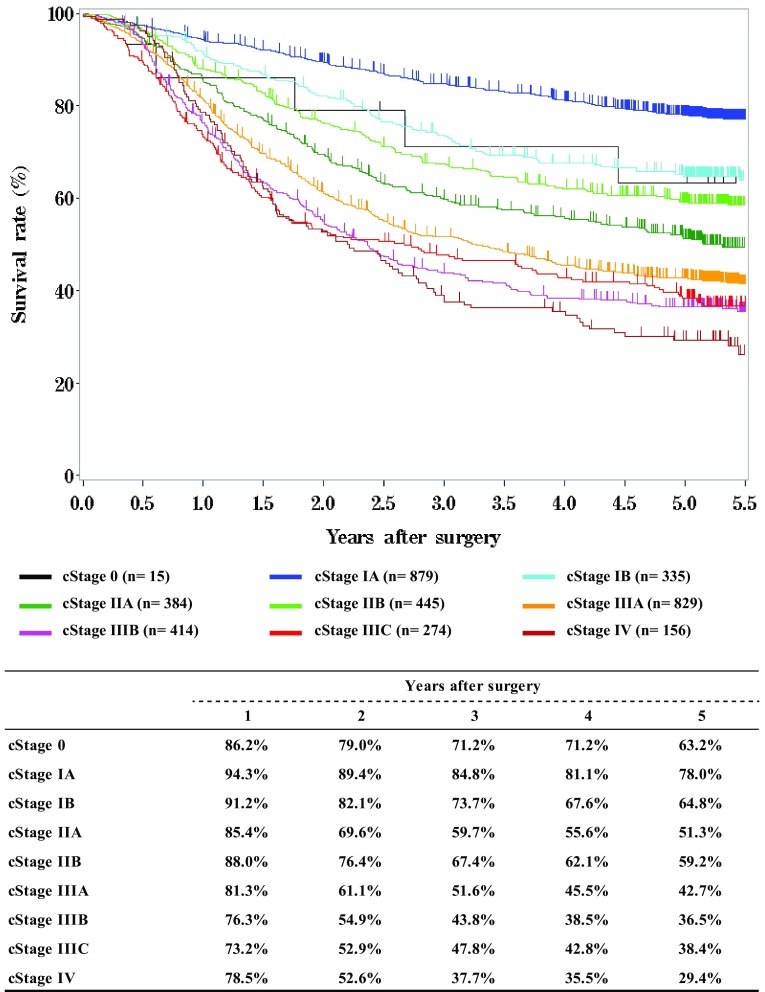
Survival of patients who underwent esophagectomy according to clinical stage (UICC 7th)

**Fig. 10:**
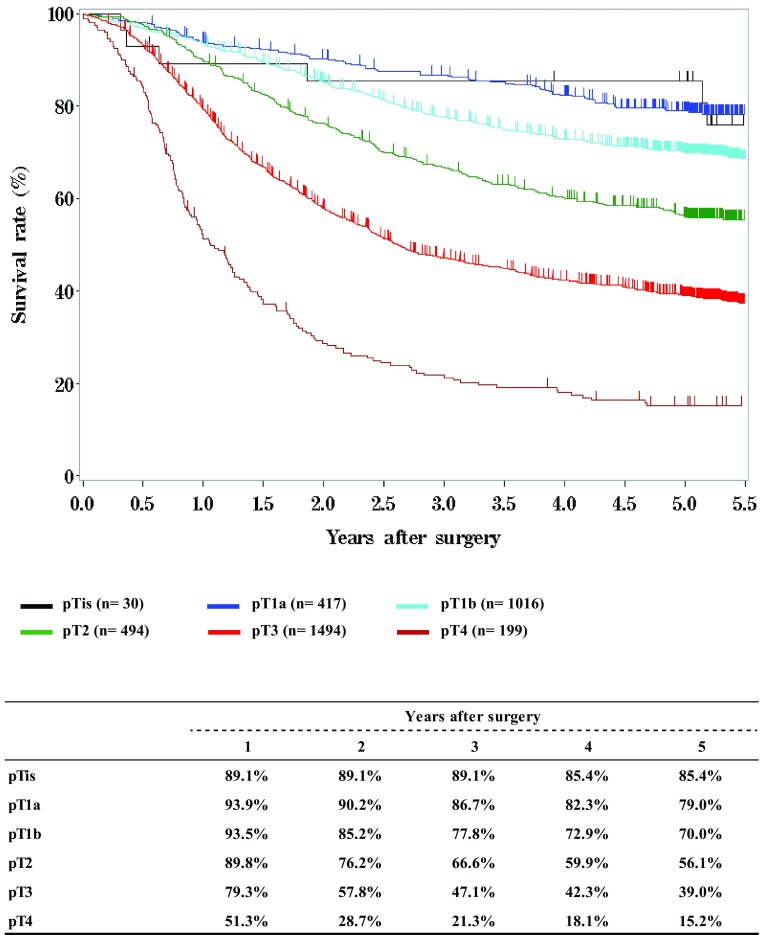
Survival of patients who underwent esophagectomy according to the depth of tumor invasion, pT (JES 10th)

**Fig. 11:**
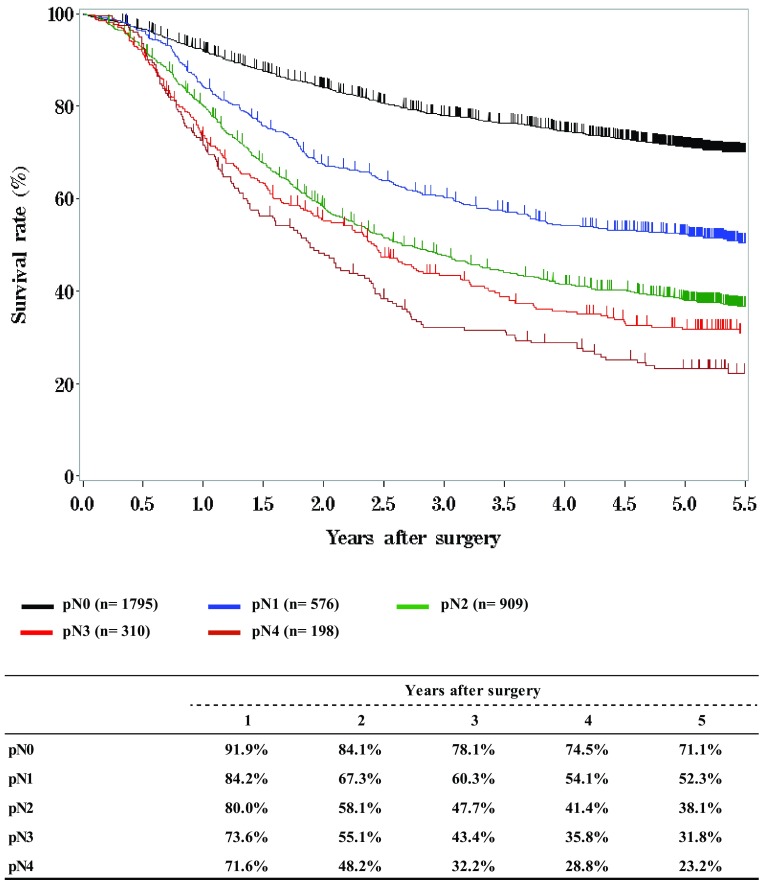
Survival of patients who underwent esophagectomy according to lymph node metastasis, pN (JES 10th)

**Fig. 12:**
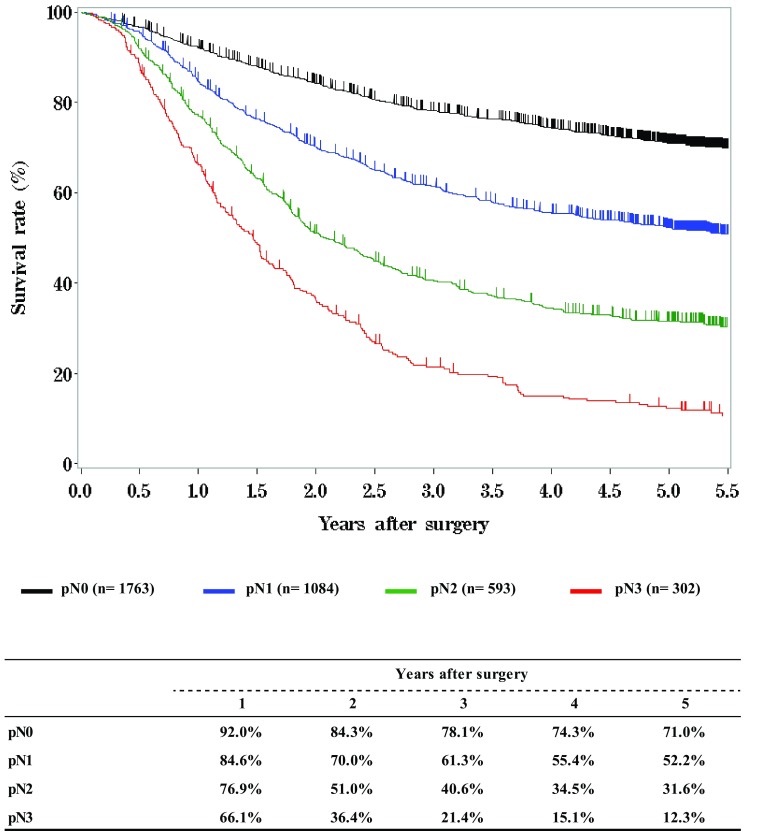
Survival of patients who underwent esophagectomy according to lymph node metastasis, pN (UICC 7th)

**Fig. 13:**
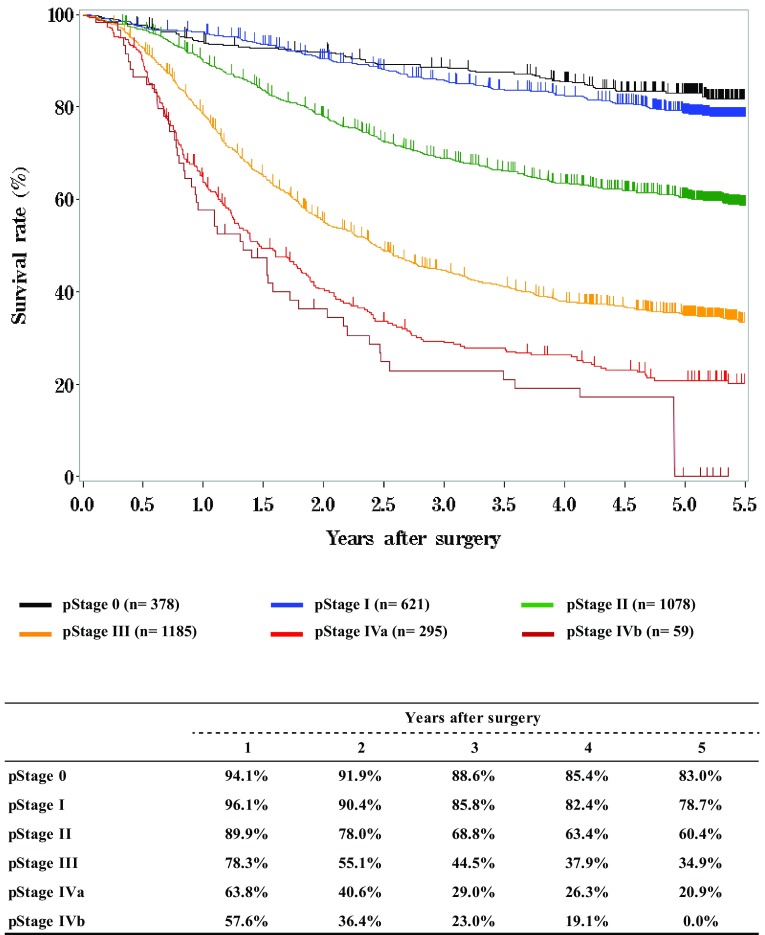
Survival of patients who underwent esophagectomy according to pathological stage (JES 10th)

**Fig. 14:**
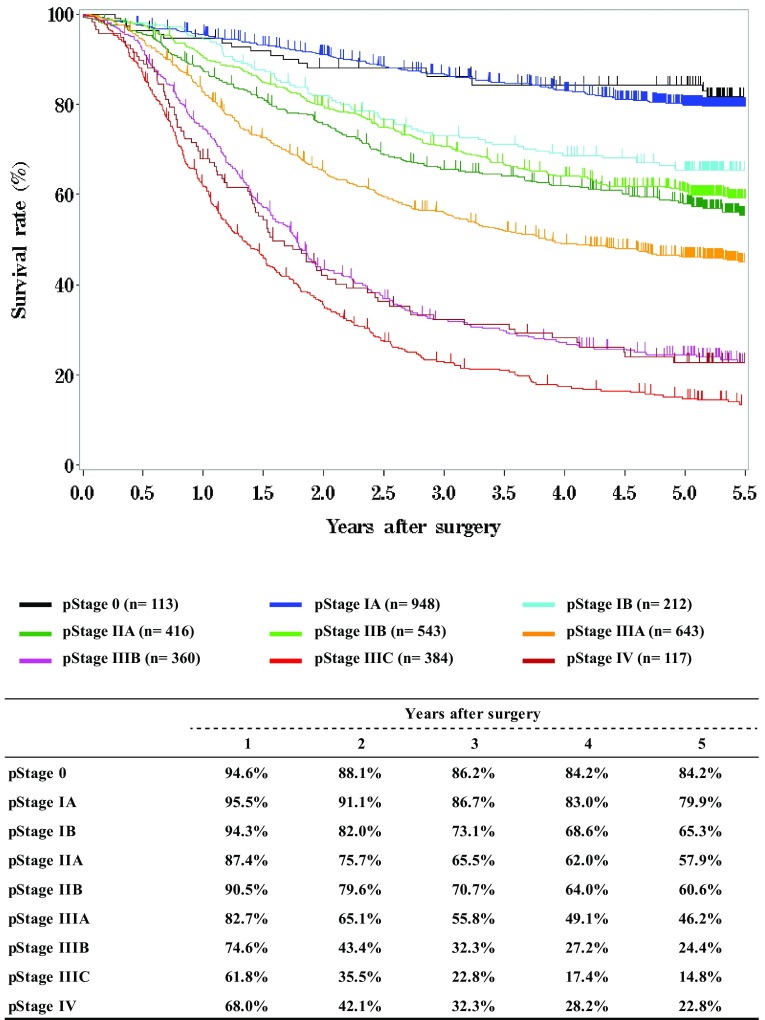
Survival of patients who underwent esophagectomy according to pathological stage (UICC TNM 7th)

**Fig. 15:**
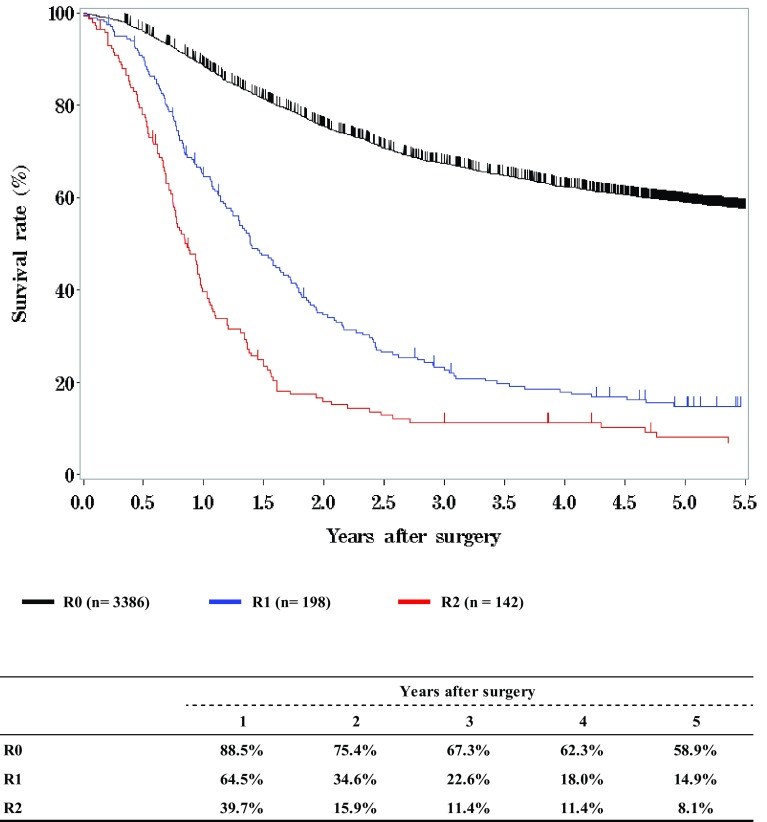
Survival of patients who underwent esophagectomy according to residual tumor (R)

I. **Clinical factors of esophageal cancer patients treated in 2011**
  1. **Institution-registered cases in 2011**
  2. **Patient background**
**Table** **1** **Age and gender**
**Table** **2** **Primary treatment**
**Table** **3** **Tumor location**
**Table** **4** **Histologic types of biopsy specimens**
**Table** **5** **Depth of tumor invasion, cT (UICC TNM 7th)**
**Table** **6** **Lymph node metastasis, cN (UICC TNM 7th)**
**Table** **7** **Distant metastasis, cM (UICC TNM 7th)**
**Table** **8** **Clinical stage (UICC TNM 7th)**
II. **Results of endoscopically treated patients in 2011**
**Table** **9** **Details of endoscopic treatment for curative intent**
**Table** **10** **Complications of EMR/ESD**
**Table** **11** **Pathological depth of tumor invasion of EMR/ESD specimens**
**Figure** **1** **Survival of patients treated with EMR/ESD**
**Figure** **2** **Survival of patients treated with EMR/ESD according to the pathological depth of tumor invasion (pT)**
**Figure** **3** **Survival of patients treated with EMR/ESD according to the lymphatic and venous invasion**
III. **Results in patients treated with chemotherapy and/or radiotherapy in 2011**
**Table** **12** **Dose of irradiation (non-surgically treated cases)**
**Table** **13** **Dose of irradiation (surgically treated cases)**
**Figure** **4** **Survival of patients treated with chemotherapy and/or radiotherapy**
**Figure** **5** **Survival of patients treated with definitive chemoradiotherapy according to clinical stage (UICC TNM 7th)**
**Figure** **6** **Survival of patients underwent radiotherapy alone according to clinical stage (UICC TNM 7th)**
IV. **Results in patients who underwent esophagectomy in 2011**
**Table** **14** **Treatment modalities of esophagectomy**
**Table** **15** **Tumor location**
**Table** **16** **Approaches to tumor resection**
**Table** **17** **Video-assisted surgery**
**Table** **18** **Fields of lymph node dissection according to the location of the tumor**
**Table** **19** **Reconstruction route**
**Table** **20** **Organs used for reconstruction**
**Table** **21** **Histological classification**
**Table** **22** **Depth of tumor invasion, pT (JES 10th)**
**Table** **23** **Pathological grading of lymph node metastasis, pN (JES 10th)**
**Table** **24** **Pathological findings of lymph node metastasis, pN (UICC 7th)**
**Table** **25** **Pathological findings of distant organ metastasis, pM (JES 10th)**
**Table** **26** **Residual tumor**
**Table** **27** **Causes of death**
**Figure** **7** **Survival of patients who underwent esophagectomy**
**Figure** **8** **Survival of patients who underwent esophagectomy according to clinical stage (JES 10th)**
**Figure** **9** **Survival of patients who underwent esophagectomy according to clinical stage (UICC 7th)**
**Figure** **10** **Survival of patients who underwent esophagectomy according to the depth of tumor invasion, pT (JES 10th)**
**Figure** **11** **Survival of patients who underwent esophagectomy according to lymph node metastasis, pN (JES 10th)**
**Figure** **12** **Survival of patients who underwent esophagectomy according to lymph node metastasis, pN (UICC 7th)**
**Figure** **13** **Survival of patients who underwent esophagectomy according to pathological stage (JES 10th)**
**Figure** **14** **Survival of patients who underwent esophagectomy according to pathological stage (UICC TNM 7th)**
**Figure** **15** **Survival of patients who underwent esophagectomy according to residual tumor (R)**

## I. Clinical factors of esophageal cancer patients treated in 2011

### Institution-registered cases in 2011

**Table:** 

Institution
Ageo Central General Hospital
Aichi Cancer Center
Aichi Medical University Hospital
Aizawa Hospital
Akita Kouseiren Hiraga Hospital
Akita University Hospital
Arao Municipal Hospital
Asahikawa Medical College Hospital
Asahikawa-Kosei General Hospital
Chiba Cancer Center
Chiba Medical Center
Chiba Prefectural Sawara Hospital
Chiba University Hospital
Chigasaki Municipal Hospital
Dokkyo Medical University Hospital
Dokkyo Medical University Saitama Medical Center
Eiju General Hospital
Foundation for Detection of Early Gastric Carcinoma
Fuchu Hospital
Fujioka General Hospital
Fujisawa Shounandai Hospital
Fujita Health University
Fukui Prefectural Hospital
Fukui University Hospital
Fukui-ken Saiseikai Hospital
Fukuoka Dental College and Dental Hospital
Fukuoka Saiseikai General Hospital
Fukuoka University Chikushi Hospital
Fukuoka University Hospital
Fukuoka Wajiro Hospital
Fukushima Medical University Hospital
Fukuyama City Hospital
Fussa Hospital
Gifu Prefectural General Medical Center
Gifu University Hospital
Gunma Central General Hospital
Gunma Prefectural Cancer Center
Gunma University Hospital
Gunmaken Saiseikai Maebashi Hospital
Hachinohe City Hospital
Hakodate Goryokaku Hospital
Hakodate National Hospital
Hamamatsu University School of Medicine, University Hospital
Hannan Chuo Hospital
Heartlife Hospital
Higashiosaka City Medical Center
Hino Memorial Hospital
Hino Municipal Hospital
Hiratsuka City Hospital
Hiratsuka Kyosai Hospital
Hirosaki University Hospital
Hiroshima City Asa Hospital
Hiroshima City Hiroshima Citizens Hospital
Hiroshima Red Cross Hospital and Atomic-bomb Survivors Hospital
Hiroshima University Hospital
Hitachi General Hospital
Hofu Institute of Gastroenterology
Hokkaido University Hospital
Hyogo Cancer Center
Hyogo College of Medicine
Hyogo Prefectural Nishinomiya Hospital
Ibaraki Prefectural Central Hospital
Iizuka Hospital
Imazu Surgical Clinic
Inazawa City Hospital
International University of Health and Welfare Hospital
International Goodwill Hospital
Isehara Kyodo Hospital
Ishikawa Prefectural Central Hospital
Iwakuni Medical Center
Iwate Medical University Hospital
Iwate Prefectural Chubu Hospital
Iwate Prefectural Isawa Hospital
Japanese Red Cross Fukui Hospital
Japanese Red Cross Ishinomaki Hospital
Japanese Red Cross Kyoto Daini Hospital
Japanese Red Cross Nagaoka Hospital
Japanese Red Cross Okayama Hospital
JCHO Kyushu Hospital
JCHO Osaka Hospital
Jichi Medical University Hospital
Jichi Medical University Saitama Medical Center
Juntendo University Hospital
Juntendo University Shizuoka Hospital
Kagawa Prefectural Central Hospital
Kagawa Rosai Hospital
Kagawa University Hospital
Kagoshima Kenritsu Satsunan Hospital
Kagoshima University Hospital
Kameda General Hospital
Kanagawa Cancer Center
Kanazawa Medical University Hospital
Kanazawa University Hospital
Kansai Medical University Hospital
Kansai Rosai Hospital
Kasamatsu Hospital
Kashiwa Kousei General Hospital
Kawasaki Medical School Hospital
Kawasaki Medical School Kawasaki Hospital
Kawasaki Municipal Ida Hospital
Nara Hospital Kinki University Faculty of Medicine
Nara Medical University Hospital
National Cancer Center Hospital
National Cancer Center Hospital East
National Center for Global Health and Medicine
National Defense Medical College Hospital
National Hospital Organization Beppu Medical Center
National Hospital Organization Chiba Medical Center
National Hospital Organization Chiba-East-Hospital
National Hospital Organization Fukuoka-higashi Medical Center
National Hospital Organization Hokkaido Cancer Center
National Hospital Organization Iwakuni Medical Center
National Hospital Organization Kanmon Medical Center
National Hospital Organization Kure Medical Center
National Hospital Organization Kyoto Medical Center
National Hospital Organization Kyushu Cancer Center
National Hospital Organization Matsumoto Medical Center
National Hospital Organization Nagasaki Medical Center
National Hospital Organization Nagoya Medical Center
National Hospital Organization Okayama Medical Center
National Hospital Organization Osaka National Hospital
National Hospital Organization Tokyo Medical Center
Niigata Cancer Center Hospital
Niigata City General Hospital
Niigata Prefectural Shibata Hospital
Niigata University Medical and Dental Hospital
Nikko Memorial Hospital
Nippon Medical School Chiba Hokusoh Hospital
Nippon Medical School Hospital
Nippon Medical School Musashi Kosugi Hospital
Nippon Medical School Tama Nagayama Hospital
Nishi-Kobe Medical Center
Nishinomiya Municipal Central Hospital
NTT WEST Osaka Hospital
Numazu City Hospital
Obihiro Kousei General Hospital
Ogaki Municipal Hospital
Ohta General Hospital Foundation Ohta Nishinouchi Hospital
Oita Red Cross Hospital
Oita University Hospital
Okayama Saiseikai General Hospital
Okayama University Hospital
Osaka City University Hospital
Osaka Hospital of Japan Seafarers relief Association
Osaka International Cancer Institute
Osaka Medical College Hospital
Osaka Police Hospital
Osaka Prefectural Hospital Organization Osaka General Medical Center
Osaka Red Cross Hospital
Osaka University Hospital
Otsu City Hospital
Otsu Red Cross Hospital
Rinku General Medical Center
Ryukyu University Hospital
Saga University Hospital
Saga-ken Medical Center Koseikan
Saiseikai Fukushima General Hospital
Saiseikai Kyoto Hospital
Saiseikai Utsunomiya Hospital
Saiseikai Yahata General Hospital
Saitama Cancer Center
Saitama City Hospital
Saitama Medical Center
Saitama Medical University Hospital
Saitama Medical University Saitama Medical Center
Sakai City Medical Center
Saku Central Hospital
Sanin Rosai Hospital
Sano Kousei General Hospital
Sato Clinic
Sendai City Hospital
Sendai Medical Center
Shiga University of Medical Science Hospital
Shikoku Cancer Center
Shimane University Hospital
Shimizu Welfare Hospital
Shin Beppu Hospital
Shinko Hospital
Shizuoka Cancer Center
Shizuoka City Shizuoka Hospital
Shizuoka General Hospital
Showa University Fujigaoka Hospital
Showa University Hospital
Showa University Koto-Toyosu Hospital
Social Insurance Omuta Tenryo Hospital
Social Insurance Tagawa Hospital
St. Marianna University School of Medical Hospital
St. Luke’s International Hospital
Sugita Genpaku Memorial Obama Municipal Hospital
Suita Municipal Hospital
Takasago Municipal Hospital
Teikyo University Chiba Medical Center
Teikyo University Hospital
Tenri Hospital
The Cancer Institute Hospital of JFCR
The Jikei University Daisan Hospital
The Jikei University Hospital
The Research Center Hospital for Charged Particle Therapy of NIRS
Tochigi Cancer Center
Toho University Ohashi Medical Center
Toho University Omori Medical Center
Toho University Sakura Medical Center
Tohoku Kosai Hospital
Tohoku University Hospital
Tokai University Hachioji Hospital
Tokai University Hospital
Tokai University Tokyo Hospital
Tokushima Red Cross Hospital
Tokushima University Hospital
Tokuyama Central Hospital
Tokyo Dental College Ichikawa General Hospital
Tokyo Medical and Dental University Hospital
Tokyo Medical University Hospital
Tokyo Medical University Ibaraki Medical Center
Tokyo Metropolitan Cancer and Infectious Diseases Center Komagome Hospital
Tokyo Metropolitan Health and Medical Corporation Toshima Hospital
Tokyo Metropolitan Tama Medical Center
Tokyo Saiseikai Central Hospital
Tokyo University Hospital
Tokyo Women’s Medical University Hospital
Tokyo Women’s Medical University Medical Center East
Tokyo Women’s Medical University Yachiyo Medical Center
Tonan Hospital
Tone Chuo Hospital
Toranomon Hospital
Tottori Prefectural Central Hospital
Tottori University Hospital
Toyama Prefectural Central Hospital
Toyama University Hospital
Toyonaka Municipal Hospital
Tsuchiura Kyodo Hospital
Tsukuba University Hospital
Tsuruoka Municipal Shonai Hospital
University Hospital, Kyoto Prefectural University of Medicine
University of Miyazaki Hospital
Urasoe General Hospital
Wakayama Medical University Hospital
Yamagata Prefectural and Sakata Municipal Hospital Organization
Yamagata Prefectural Central Hospital
Yamagata Prefectural Shinjo Hospital
Yamagata University Hospital
Yamaguchi University Hospital
Yamaguchi-ken Saiseikai Shimonoseki General Hospital
Yamanashi Prefectural Central Hospital
Yamanashi University Hospital
Yao Municipal Hospital
Yokohama Chuo Hospital
Yokohama City Municipal Hospital
Yokohama City University Medical Center
Yokohama Rosai Hospital

(Total 300 institutions)

### Patient background

**Table 1:** Age and gender

Age	Male	Female	Cases (%)
≤ 29	4	1	5 (0.1%)
30 – 39	22	8	30 (0.4%)
40 – 49	142	47	189 (2.7%)
50 – 59	878	173	1051 (15.0%)
60 – 69	2531	360	2891 (41.3%)
70 – 79	1941	333	2274 (32.5%)
80 – 89	442	90	532 (7.6%)
90–	13	8	21 (0.3%)
Total	5973	1020	6993

**Table 2:** Primary treatment

Treatments	Cases (%)
Surgery	4236 (60.7%)
Esophagectomy	4147 (59.4%)
Palliative surgery	89 (1.3%)
Chemotherapy/radiotherapy	1549 (22.2%)
Endoscopic treatment	1198 (17.2%)
Total	6983

**Table 3:** Tumor location

Location of tumor	Endoscopic treatment (%)	Surgery		Chemotherapy and/or radiotherapy (%)	Total (%)
		Esophagectomy (%)	Palliative surgery (%)		
Cervical	33 (2.8%)	127 (3.1%)	4 (4.5%)	147 (9.5%)	311 (4.5%)
Upper thoracic	116 (9.7%)	517 (12.5%)	18 (20.2%)	256 (16.5%)	907 (13.0%)
Middle thoracic	687 (57.3%)	1873 (45.2%)	46 (51.7%)	732 (47.3%)	3338 (47.8%)
Lower thoracic	296 (24.7%)	1235 (29.8%)	20 (22.5%)	345 (22.3%)	1896 (27.2%)
EG	41 (3.4%)	300 (7.2%)	0	36 (2.3%)	377 (5.4%)
E = G	9 (0.8%)	47 (1.1%)	0	1 (0.1%)	57 (0.8%)
GE	5 (0.4%)	40 (1.0%)	1 (1.1%)	2 (0.1%)	48 (0.7%)
Unknown	11 (0.9%)	8 (0.2%)	0	30 (1.9%)	49 (0.7%)
Total	1198	4147	89	1549	6983

*E* esophageal, *G* gastric

**Table 4:** Histologic types of biopsy specimens

Histologic types	Cases (%)
Squamous cell carcinoma	6164 (88.3%)
Squamous cell carcinoma	4369 (62.6%)
Well differentiated	378 (5.4%)
Moderately differentiated	1054 (15.1%)
Poorly differentiated	363 (5.2%)
Adenocarcinoma	281 (4.0%)
Barrett’s adenocarcinoma	90 (1.3%)
Adenosquamous carcinoma	15 (0.2%)
Mucoepidermoid carcinoma	4 (0.1%)
Basaloid carcinoma	35 (0.5%)
Neuroendocrine cell tumor	26 (0.4%)
Undifferentiated carcinoma	8 (0.1%)
Sarcoma	6 (0.1%)
Malignant melanoma	19 (0.3%)
Carcinosarcoma	22 (0.3%)
GIST	7 (0.1%)
Other tumors	92 (1.3%)
Unknown	214 (3.1%)
Total	6983

**Table 5:** Depth of tumor invasion, cT (UICC TNM 7th)

cT	Cases (%)
cTX	71 (1.0%)
cT0	10 (0.1%)
cTis	198 (2.8%)
cT1a	1051 (15.1%)
cT1b	1292 (18.5%)
cT2	905 (13.0%)
cT3	2408 (34.5%)
cT4a	384 (5.5%)
cT4b	530 (7.6%)
Unknown	134 (1.9%)
Total	6983

**Table 6:** Lymph node metastasis, cN (UICC TNM 7th)

cN	Cases (%)
cNX	187 (2.7%)
cN0	3195 (45.8%)
cN1	1864 (26.7%)
cN2	1199 (17.2%)
cN3	459 (6.6%)
Unknown	79 (1.1%)
Total	6983

**Table 7:** Distant metastasis, cM (UICC TNM 7th)

cM	Cases (%)
cM0	6128 (87.8%)
cM1	722 (10.3%)
Unknown	133 (1.9%)
Total	6983

**Table 8:** Clinical stage (UICC TNM 7th)

Clinical stage	Endoscopic treatment (%)	Surgery		Chemotherapy and/or radiotherapy (%)	Total (%)
		Esophagectomy (%)	Palliative surgery (%)		
Stage 0	151 (12.6%)	15 (0.4%)	0	7 (0.5%)	173 (2.5%)
Stage IA	809 (67.5%)	937 (22.6%)	1 (1.1%)	161 (10.4%)	1908 (27.3%)
Stage IB	2 (0.2%)	363 (8.8%)	1 (1.1%)	58 (3.7%)	424 (6.1%)
Stage IIA	3 (0.3%)	419 (10.1%)	3 (3.4%)	60 (3.9%)	485 (6.9%)
Stage IIB	4 (0.3%)	470 (11.3%)	1 (1.1%)	63 (4.1%)	538 (7.7%)
Stage IIIA	10 (0.8%)	898 (21.7%)	14 (15.7%)	147 (9.5%)	1069 (15.3%)
Stage IIIB	6 (0.5%)	456 (11.0%)	9 (10.1%)	99 (6.4%)	570 (8.2%)
Stage IIIC	32 (2.7%)	292 (7.0%)	27 (30.3%)	390 (25.2%)	741 (10.6%)
Stage IV	40 (3.3%)	165 (4.0%)	25 (28.1%)	434 (28.0%)	664 (9.5%)
Unknown	141 (11.8%)	132 (3.2%)	8 (9.0%)	130 (8.4%)	411 (5.9%)
Total	1198	4147	89	1549	6983

## II. Results of endoscopically treated patients in 2011

**Table 9:** Details of endoscopic treatment for curative intent

Treatment details	Cases (%)
EMR	190 (17.9%)
EMR + YAG laser	13 (1.2%)
ESD	829 (78.1%)
ESD + EMR	5 (0.5%)
ESD + PDT	0
ESD + YAG laser	5 (0.5%)
PDT	2 (0.2%)
YAG laser	18 (1.7%)
Total	1062

*EMR* endoscopic mucosal resection, *ESD* endoscopic submucosal dissection, *YAG* yttrium aluminum garnet, *PDT* photodynamic therapy

**Table 10:** Complications of EMR/ESD

Complications of EMR/ESD	Cases (%)
None	969 (93.0%)
Perforation	13 (1.2%)
Bleeding	3 (0.3%)
Mediastinitis	3 (0.3%)
Stenosis	49 (4.7%)
Others	4 (0.4%)
Total	1042

**Table 11:** Pathological depth of tumor invasion of EMR/ESD specimens

Pathological depth of tumor invasion (pT)	Cases (%)
pTX	3 (0.3%)
pT0	7 (0.7%)
pTis	201 (19.3%)
pT1a	703 (67.5%)
pT1b	114 (10.9%)
pT2	3 (0.3%)
Unknown	11 (1.1%)
Total	1042

## III. Results in patients treated with chemotherapy and/or radiotherapy in 2011

**Table 12:** Dose of irradiation (non-surgically treated cases)

Dose of irradiation (Gy)	Definitive		Palliative (%)	Recurrence (%)	Others (%)	Unknown (%)	Total (%)
	Radiation alone (%)	Chemoradiotherapy (%)					
− 29	6 (3.5%)	13 (1.7%)	32 (10.9%)	0	2 (5.7%)	0	53 (4.1%)
30–39	4 (2.3%)	17 (2.2%)	40 (13.6%)	0	2 (5.7%)	0	63 (4.9%)
40–49	8 (4.6%)	33 (4.2%)	34 (11.6%)	0	10 (28.6%)	0	85 (6.6%)
50–59	29 (16.8%)	177 (22.7%)	71 (24.1%)	1 (25.0%)	11 (31.4%)	1 (50.0%)	290 (22.5%)
60–69	116 (67.1%)	516 (66.1%)	108 (36.7%)	3 (75.0%)	9 (25.7%)	0	752 (58.3%)
70–	9 (5.2%)	12 (1.5%)	3 (1.0%)	0	1 (2.9%)	0	25 (2.2%)
Unknown	1 (0.6%)	13 (1.7%)	6 (2.0%)	0	0	1 (50.0%)	21 (1.6%)
Total	173	781	294	4	35	2	1289
Median (min–max)	60.0 (4.4–70.0)	60.0 (1.8–120.0)	50.4 (3.6–159.0)	60.0 (50.0–61.2)	50.0 (21.6–109.0)	54.0 (54.0–54.0)	60.0 (1.8–105.0)

**Table 13:** Dose of irradiation (surgically treated cases)

Dose of irradiation (Gy)	Preoperative irradiation (%)	Postoperative irradiation (%)
–29	5 (2.2%)	3 (6.0%)
30–39	39 (17.1%)	0
40–49	156 (68.4%)	8 (16.0%)
50–59	15 (6.6%)	15 (30.0%)
60–69	6 (2.6%)	15 (30.0%)
70–	1 (0.4%)	0
Unknown	6 (2.6%)	9 (18.0%)
Total	228	50
Median (min–max)	40.0 (1.8–70.0)	50.4 (2.0–66.0)

## IV. Results in patients who underwent esophagectomy in 2011

**Table 14:** Treatment modalities of esophagectomy

Treatments	Cases (%)
Esophagectomy alone	1699 (41.2%)
Esophagectomy + endoscopic treatment	89 (2.2%)
Esophagectomy + chemoradiotherapy	590 (14.3%)
Concurrent chemoradiotherapy	370 (9.0%)
Other	220 (5.3%)
Esophagectomy + chemoradiotherapy + endoscopic treatment	21 (0.5%)
Esophagectomy + chemotherapy	1657 (40.2%)
Preoperative	1295 (31.4%)
Postoperative	198 (4.8%)
Preoperative and postoperative	57 (1.4%)
Recurrence	107 (2.6%)
Other	20 (0.5%)
Esophagectomy + chemotherapy + endoscopic treatment	1 (0.0%)
Esophagectomy + radiotherapy	67 (1.6%)
Preoperative	17 (0.4%)
Postoperative	13 (0.3%)
Recurrence	5 (0.1%)
Other	32 (0.8%)
Esophagectomy + radiotherapy + endoscopic treatment	3 (0.1%)
Total	4127

**Table 15:** Tumor location

Locations	Cases (%)
Cervical	127 (3.1%)
Upper thoracic	517 (12.5%)
Middle thoracic	1873 (45.2%)
Lower thoracic	1235 (29.8%)
E > G	300 (7.2%)
E = G	47 (1.1%)
G > E	40 (1.0%)
Unknown	8 (0.2%)
Total lesions	4147

**Table 16:** Approaches to tumor resection

Approaches	Cases (%)
Cervical approach	96 (2.3%)
Right thoracic	3459 (83.4%)
Left thoracic	67 (1.6%)
Left thoracoabdominal	72 (1.7%)
Abdominal	172 (4.1%)
Transhiatal thoracic esophagectomy	51 (1.2%)
Transhiatal lower esophagectomy	82 (2.0%)
Sternotomy	9 (0.2%)
Others	33 (0.8%)
Unknown	106 (2.6%)
Total	4147

Thoracic includes thoracotomy and thoracoscopic. Abdominal includes laparotomy and laparoscopic

**Table 17:** Video-assisted surgery

Video-assisted surgery	Cases (%)
None	2389 (57.6%)
Thoracoscopy	768 (18.5%)
Thoracoscopy + Laparoscopy	605 (14.6%)
Thoracoscopy + Laparoscopy + Mediastinoscopy	15 (0.4%)
Thoracoscopy + Mediastinoscopy	2 (0.0%)
Laparoscopy	201 (4.8%)
Laparoscopy + Mediastinoscopy	14 (0.3%)
Laparoscopy + Other	2 (0.0%)
Mediastinoscopy	21 (0.5%)
Others	4 (0.1%)
Total	4147

**Table 18:** Fields of lymph node dissection according to the location of the tumor

Field of lymphadenectomy	Cervical	Upper thoracic	Middle thoracic	Lower thoracic	E > G	E = G	G > E	Unknown	Total
None	10 (8.6%)	13 (3.2%)	59 (3.5%)	28 (2.7%)	13 (5.4%)			2 (25.0%)	125 (3.5%)
C	36 (31.0%)	10 (2.4%)	20 (1.2%)	3 (0.3%)	1 (0.4%)				70 (2.0%)
C + UM	21 (18.1%)	6 (1.5%)	3 (0.2%)	1 (0.1%)					31 (0.9%)
C + UM + MLM	2 (1.7%)	12 (2.9%)	28 (1.7%)	12 (1.1%)			1 (3.7%)		55 (1.5%)
C + UM + MLM + A	27 (23.3%)	257 (62.5%)	800 (47.9%)	367 (34.8%)	26 (10.8%)	6 (15.8%)		1 (12.5%)	1484 (41.6%)
C + UM + MLM + A+OT				1 (0.1%)					1 (0.0%)
C + UM + A	2 (1.7%)	1 (0.2%)	2 (0.1%)	2 (0.2%)					7 (0.2%)
C + MLM			1 (0.1%)						1 (0.0%)
C + MLM + A	3 (2.6%)	1 (0.2%)	7 (0.4%)	3 (0.3%)					14 (0.4%)
C + A	1 (0.9%)	2 (0.5%)	4 (0.2%)	2 (0.2%)	1 (0.4%)				10 (0.3%)
UM	4 (3.4%)	3 (0.7%)	5 (0.3%)	3 (0.3%)					15 (0.4%)
UM + MLM	1 (0.9%)	7 (1.7%)	29 (1.7%)	12 (1.1%)	1 (0.4%)			1 (12.5%)	51 (1.4%)
UM + MLM + A	3 (2.6%)	75 (18.2%)	627 (37.6%)	478 (45.4%)	56 (23.2%)	5 (13.2%)	1 (3.7%)	1 (12.5%)	1246 (35.0%)
UM + A	1 (0.9%)	4 (1.0%)	2 (0.1%)	2 (0.2%)	2 (0.8%)				11 (0.3%)
MLM		3 (0.7%)	10 (0.6%)	14 (1.3%)	3 (1.2%)				30 (0.8%)
MLM + A	1 (0.9%)	7 (1.7%)	34 (2.0%)	102 (9.7%)	108 (44.8%)	23 (60.5%)	17 (63.0%)		292 (8.2%)
A	1 (0.9%)	6 (1.5%)	22 (1.3%)	12 (1.1%)	28 (11.6%)	3 (7.9%)	8 (29.6%)	1 (12.5%)	81 (2.3%)
Unknown	3 (2.6%)	4 (1.0%)	16 (1.0%)	12 (1.1%)	2 (0.8%)	1 (2.6%)		2 (25.0%)	40 (1.1%)
Total	116	411	1669	1054	241	38	27	8	3564

*C* bilateral cervical nodes, *UM* upper mediastinal nodes, *MLM* middle–lower mediastinal nodes, *A* abdominal nodes

**Table 19:** Reconstruction route

Reconstruction route	Cases (%)
None	56 (1.4%)
Subcutaneous	384 (9.3%)
Retrosternal	1437 (34.7%)
Posterior mediastinal	1715 (41.4%)
Intrathoracic	419 (10.1%)
Cervical	35 (0.8%)
Others	34 (0.8%)
Unknown	67 (1.6%)
Total	4147

**Table 20:** Organs used for reconstruction

Organs used for reconstruction	Cases (%)
None	76 (1.8%)
Whole stomach	63 (1.5%)
Gastric tube	3508 (83.6%)
Jejunum	255 (6.1%)
Free jejunum	76 (1.8%)
Colon	127 (3.0%)
Free colon	13 (0.3%)
Skin graft	1
Others	14 (0.3%)
Unknown	63 (1.5%)
Total organs	4196
Total cases	4147

**Table 21:** Histological classification

Histological classification	Cases (%)
Squamous cell carcinoma	3502 (84.4%)
Squamous cell carcinoma	732 (17.7%)
Well differentiated	645 (15.6%)
Moderately differentiated	1630 (39.3%)
Poorly differentiated	495 (11.9%)
Adenocarcinoma	210 (5.1%)
Barrett’s adenocarcinoma	78 (1.9%)
Adenosquamous carcinoma	31 (0.7%)
Mucoepidermoid carcinoma	3 (0.1%)
Adenoid cystic carcinoma	2 (0.0%)
Basaloid carcinoma	81 (2.0%)
Neuroendocrine cell tumor	15 (0.4%)
Undifferentiated carcinoma	8 (0.2%)
Other carcinoma	9 (0.2%)
Carcinosarcoma	29 (0.7%)
Malignant melanoma	16 (0.4%)
GIST	6 (0.1%)
Other	39 (0.9%)
Unknown	118 (2.8%)
Total	4147

**Table 22:** Depth of tumor invasion, pT (JES 10th)

pT category	Cases (%)
pTX	57 (1.4%)
pT0	128 (3.1%)
pTis	31 (0.7%)
pT1a	435 (10.5%)
pT1b	1070 (25.8%)
pT2	516 (12.4%)
pT3	1576 (38.0%)
pT4	24 (0.6%)
pT4a	93 (2.2%)
pT4b	89 (2.1%)
Unknown	128 (3.1%)
Total	4147

**Table 23:** Pathological grading of lymph node metastasis, pN (JES 10th)

Lymph node metastasis	Cases (%)
pN0	1970 (47.5%)
pN1	616 (14.9%)
pN2	949 (22.9%)
pN3	323 (7.8%)
pN4	209 (5.0%)
Unknown	80 (1.9%)
Total	4147

**Table 24:** Pathological findings of lymph node metastasis, pN (UICC 7th)

Lymph node metastasis	Cases (%)
pN0	1871 (45.1%)
pN1 (1–2)	1165 (28.1%)
pN2 (3–6)	659 (15.9%)
pN3 (7–)	366 (8.8%)
Unknown	86 (2.1%)
Total	4147

Regional lymph nodes are different in JES 10th and UICC 7th

Data for Tables 23 and 24 were analyzed from different variables in the registration application

**Table 25:** Pathological findings of distant organ metastasis, pM (JES 10th)

Distant metastasis	Cases (%)
pMX	195 (4.7%)
pM0	3886 (93.7%)
pM1	66 (1.6%)
Total	4147

**Table 26:** Residual tumor

Residual tumor	Cases (%)
RX	147 (3.5%)
R0	3624 (87.4%)
R1	219 (5.3%)
R2	157 (3.8%)
Total	4147

**Table 27:** Causes of death

Cause of death	Cases (%)
Death due to recurrence	1223 (71.2%)
Death due to other cancer	71 (4.1%)
Death due to other disease (rec+)	42 (2.4%)
Death due to other disease (rec−)	239 (13.9%)
Death due to other disease (rec?)	9 (0.5%)
Operative death*	27 (1.6%)
Postoperative hospital death**	55 (3.2%)
Unknown	51 (3.0%)
Total of death cases	1717

rec: recurrence

*Operative death means death within 30 days after operation in or out of hospital

**Hospital death is defined as death during the same hospitalization, regardless of department at time of death

Operative mortality after esophagectomy: 0.65%

Hospital mortality after esophagectomy: 3.76%

